# Multi-basis-image reconstruction from conventional data acquired in standard CT

**DOI:** 10.1088/1361-6560/add789

**Published:** 2025-05-22

**Authors:** Buxin Chen, Zheng Zhang, Dan Xia, Emil Y Sidky, Xiaochuan Pan

**Affiliations:** 1 Department of Radiology, The University of Chicago, Chicago, IL 60637, United States of America; 2 Department of Radiation and Cellular Oncology, The University of Chicago, Chicago, IL 60637, United States of America

**Keywords:** multi-basis image, conventional CT, basis regions, volume-conservation constraint

## Abstract

*Objective.* We investigate and develop an algorithm to invert the well-established non-linear data model in standard computed tomography (CT) for numerically accurate and stable reconstruction of multi ($\unicode{x2A7E} 2$)-basis images directly from a set of conventional data collected with a single spectrum in standard CT. *Approach.* Using the basis-region technique to reduce the number of voxel values, i.e. unknowns, in the basis images to be reconstructed and the volume-conservation constraint to augment conventional data, we formulate the reconstruction problem (i.e. the inverse problem) as a non-convex optimization program and develop the dynamic non-convex primal-dual (dNCPD) algorithm to empirically solve the optimization program for numerically accurate and stable reconstruction of multi-basis images from conventional data. *Main results.* We conduct studies to verify numerically the reconstruction accuracy of the dNCPD algorithm with simulated conventional data and also studies to evaluate the stability of the dNCPD algorithm with real conventional data that contain noise and other physical factors. The study results reveal that the dNCPD algorithm can numerically accurately and stably yield multi-basis images and virtual monochromatic images from conventional data. *Significance.* The work can be of theoretic interest and practical implication as it reveals the possibility of yielding multi-basis images from conventional data in standard CT, instead of data collected in dual-energy, multi-spectra, or photon-counting CT.

## 1. Introduction

In standard computed tomography (CT), the polychromatic nature of the x-rays involved leads to a non-linear data model that relates data collected to the image, i.e. the spatial distribution of linear attenuation coefficient (LAC) within the subject of interest. When the reconstruction of an image from CT data is based upon a linear model, it results in beam-hardening (BH) artifacts that may plague the visualization, and bias the quantification, of the image reconstructed. Furthermore, as the subject is composed generally of multi-basis materials, it can thus be of practical interest in reconstructing the spatial distributions of the basis materials, i.e. basis images.

The current approach is to reconstructing multi ($\unicode{x2A7E} \!\!2$)-basis images, which may also address the issue of BH correction, from two sets of data collected with two distinct spectra in dual-energy CT (DECT), which are referred to simply as dual-energy (DE) data hereinafter. Image-domain-decomposition and data-domain-decomposition methods are used for obtaining basis images from DE data (Johnson 2007, Granton *et al* 2008, Zou and Silver 2008, Liu *et al* 2009, Maass *et al* 2009). Additionally, optimization-based algorithms have been investigated for reconstruction of two- or multi ($\unicode{x2A7E} \!\!2$)-basis images directly from DE data (Cai *et al* 2013, Long and Fessler 2014, Zhao *et al* 2014, Mechlem *et al* 2017, Liu *et al* 2022) through inverting the non-linear data model.

The collection of DE data with two distinct spectra (Tang 2023) can generally increase the scanning effort and time and/or may require additional hardware such as two pairs of sources and detectors (Flohr 2006), or a specialized x-ray tube capable of slow- or fast-kV switching (Xu *et al* 2009), or two layer (i.e. sandwiched) detectors (Carmi *et al* 2005). While the radiation dose to the subject imaged can be elevated with two separate scans, additional hardware required may also add cost to its acquisition and maintenance. Therefore, it is of theoretical interest and practical significance to investigate algorithms that can accurately reconstruct multi-basis images, and also correct for the BH effect, from conventional data collected with a single spectrum in a standard CT scan. Conventional data collected with a single spectrum in standard CT are referred to often as single-energy (SE) data, as opposed to DE data collected in DECT; and we simply refer to them as conventional data hereinafter in this work to avoid possible confusion with data collected using true SE x-rays, e.g. in synchrotron imaging.

While two-step methods have been investigated for approximately reconstructing basis images from conventional data through material decomposition in either the image domain (Xue *et al* 2020) or the data domain (Kis *et al* 2012), there is no evidence that the two-step methods can numerically accurately invert the non-linear data model for yielding multi-basis images. In recent years, works have been reported that use learning-based neural network methods (Lyu *et al* 2021, Li *et al* 2023) for reconstructing two basis images from conventional data. It appears that the learning-based methods require a considerable number of high-quality training images reconstructed from DE data by use of existing image- or data-domain-decomposition methods and that some of the methods may also, in addition to conventional data, require partial data collected with a high-energy spectrum (Lyu *et al* 2021). Furthermore, it remains unclear if the learning-based neural network methods can indeed invert the non-linear data model for achieving numerically accurate reconstruction of multi-basis images and correction for the BH effect. The learning-based methods are trained to yield only two basis images even if the subject contains multi-basis materials.

In the work, we investigate a one-step algorithm, referred to as the dynamic non-convex primal-dual (dNCPD) algorithm, for directly inverting the non-linear data model. A key novelty of the work lies in numerically accurately and stably solving the non-convex optimization programs and thus accurately reconstructing multi-basis images from conventional data collected in standard CT.

The inverse problem, i.e. reconstruction problem of multi-basis images, from conventional data can be considerably ill-posed as the result of limited information contained in conventional data in standard CT. In an attempt to alleviate the degree of ill-posedness of the inverse problem, we thus exploit, on one hand, the basis-region technique (Chen *et al* 2024) for reducing the number of unknowns involved in basis images and, on the other hand, the volume-conservation (VC) constraint (Goodsitt *et al* 1987, Mendonca *et al* 2013) for augmenting conventional data. Using the technique and constraint, we formulate the inverse problem, i.e. the reconstruction problem of multi-basis images, as a non-convex optimization program; and we subsequently develop the dNCPD algorithm to solve the optimization program empirically for achieving numerically accurate reconstruction of multi-basis images from conventional data.

We perform numerical studies with simulation data of digital phantoms to verify that the dNCPD algorithm developed and its computer implementation can numerically accurately invert the non-linear data model and reconstruct multi-basis images from conventional data. Furthermore, we conduct studies using real conventional data collected from physical and clinical phantoms of relevance to evaluate the stability of the dNCPD algorithm in reconstruction of multi-basis images and virtual monochromatic images (VMIs).

We present the development of the dNCPD algorithm in section 2, the numerical studies on verifying the accuracy, and evaluating the stability, of the dNCPD algorithm in section 3, and discussion and conclusion of the work in sections 4 and 5.

## 2. Methods

To facilitate the development of the dNCPD algorithm, we first summarize the well-established non-linear data model with the x-ray polychromaticity included in standard CT. We use $g_{j}(\mathbf{b})$ to denote model data of ray *j*, where $j = 1,2, {\ldots}, J$ and *J* the total number of rays involved; $q_{j{m}}$ the normalized effective spectrum for ray *j* at energy bin *m*, where $m = 1,2, {\ldots}, M$ and *M* the total number of energy bins; and $\mu_{k {m}}$ the mass attenuation coefficient at *m* of material *k*, where $k = 1,2,\cdots, K$ and *K* the total number of basis-material types. Let vector **b**_*k*_ of size *I* denote basis image *k* on the full image array of size *I* with entries *b*_*ki*_, where $i = 1, 2, \cdots, I$. For discussion convenience, we use vector **b** of size $K I$ to denote congregated basis image obtained by concatenating **b**_*k*_ in the order of *k*.

In standard CT, we can express the well-established non-linear data model in a discrete-to-discrete form as

\begin{equation*} \begin{aligned} g_{j}\left(\mathbf{b}\right) &amp; = - \ln \sum_{m = 1}^{M} q_{j{m}} \exp \left( - \mathbf{A}_j^\top \sum_{k = 1}^K \mu_{k m} \mathbf{b}_k \right), \end{aligned}\end{equation*}

where vector **A**_*j*_ of size *I* denotes row *j* of matrix $\mathcal{A}$ of size *J* × *I*, which depicts the discrete x-ray transform, with element *a*_*ji*_ representing the weight of the contribution of voxel *i* (i.e. column *i*) to model data of ray *j* (i.e. row *j*); and *$\top$* indicates the transpose operation. It can be observed in data model equation (1) that basis images **b**_*k*_ of interest are related non-linearly to model data $g_{j}(\mathbf{b})$ for polychromatic spectrum $q_{j{m}}$. For a monochromatic spectrum, i.e. *q*_*jm*_ has only one non-zero value among all energy bins, basis images **b**_*k*_ are related then linearly to model data $g_{j}(\mathbf{b})$.

While it is possible that $K\unicode{x2A7E} 3$ basis images **b**_*k*_ can be accurately and stably reconstructed directly from DE data collected with two distinct spectra (Chen *et al* 2024), we seek instead in this work to develop algorithms to invert the non-linear data model in equation (1) for numerically accurate and stable reconstruction of $K\unicode{x2A7E} 3$ basis images **b**_*k*_ directly from conventional data collected with a single spectrum in standard CT. As discussed above, the inverse problem from conventional data is generally highly ill-posed as conventional data contain an amount of information significantly less than that in DE data.

### 2.1. Improving the well-posedness of the data model

In an attempt to alleviate the degree of ill-posedness of the inverse problem from conventional data, we thus exploit, on one hand, the basis-region technique (Chen *et al* 2024) for reducing the number of unknowns involved in the reconstruction of multi-basis images and, on the other hand, the VC constraint (Goodsitt *et al* 1987) for augmenting conventional data.

#### 2.1.1. Reduction of unknowns with basis-region images

##### 2.1.1.1. Basis regions and basis-region images

As the basis materials of the subject imaged are often confined within spatial regions smaller than the full image array, we can partition the full image array into *L* spatially complementary basis regions, and assume that basis region *l*, where $l = 1, 2, \cdots, L$, contains *K_l_* known types of basis materials (Chen *et al* 2024). In this work, we specifically assume that each of the basis regions contains $K_l\unicode{x2A7D} 2$ known types of basis materials.

Let $\mathcal{R}_l$ denote a diagonal matrix of size *I* × *I*, specifying basis region *l* inside or outside which voxel values are 1 or 0, respectively, and $\Phi_l$ denote a set of indices *k*’s of basis-material types contained in basis region *l*. Basis region *l* can then be completely characterized by $\mathcal{R}_l$ and $\Phi_l$. Using vector $\mathbf{b}^{^{\prime}}_{lk}$ of size $I_l < I$, referred to as the basis-region image, to denote the spatial distribution of basis material *k* within basis region *l*, we have $I_l = \mathrm{tr}(\mathcal{R}_l) < I$ and $K_l = \mathrm{card}(\Phi_l) \unicode{x2A7D} K $, which are the total number of voxels of basis-region image $\mathbf{b}^{^{\prime}}_{lk}$ and the total number of basis-material types contained in basis region *l*.

**Figure 1.**
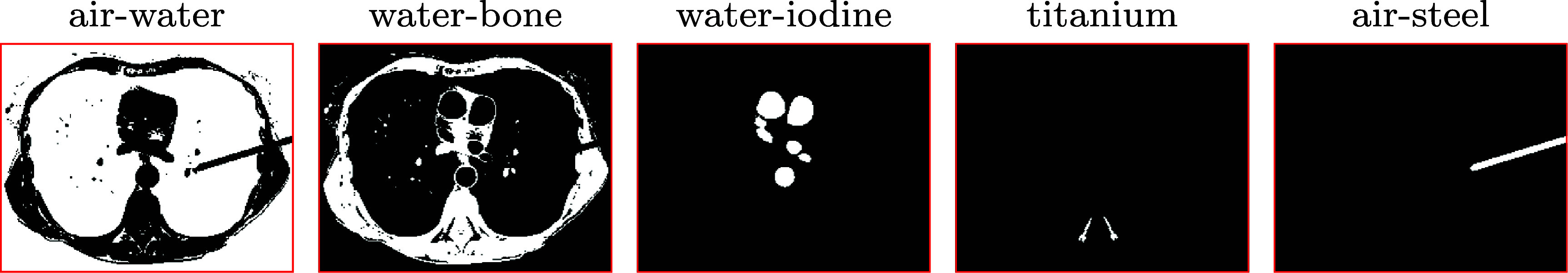
Truth basis regions, highlighted in white, of the digital chest phantom are displayed on the full image array. Clearly, the number of active pixels within each of the basis regions is smaller than the number of pixels within the full image array. The truth basis regions are used for generation of simulated conventional data and for reconstruction of basis images in the verification study. Each basis region contains up to 2 basis materials, including air and water (column 1), water and bone (column 2), water and 20 mg ml^−1^ iodine solution (column 3), titanium (column 4), and air and stainless steel (column 5), respectively.

We have considered two rules of selecting basis regions. First, a set of basis regions must be spatially complementary and form a partition of the full image array. The active pixels (highlighted in white) in each basis region do not overlap with those in another basis region, and the union of the active pixels from all basis regions forms the full image array (see figures 1, 4, and 8 below.) In other words, each pixel in the full image array belongs to one and only one of the basis regions. Second, each basis region must have a unique set of basis materials. That is, each basis region contains a unique subset of the full list of basis materials. Within a basis region, only those basis materials that are in its subset (i.e. contained in the basis region) can have non-zero pixel values and be updated from the data in reconstruction. On the contrary, those basis materials that are not in the subset (i.e. not contained in the basis region) remain zeros within the basis region. In this work, in particular, it is assumed that there are at most two basis materials in each basis region. Therefore, the unique subset of basis materials for each basis region has at most two elements. Therefore, a basis region is a spatial region with a reduced number of pixels that contains a unique list of basis materials. The initialization of basis regions often involves the reconstruction from the conventional data and identifying the basis materials contained in the image, as well as their distributions.

##### 2.1.1.2. Basis-region images, basis images, and VMIs

We can write basis images **b**_*k*_ on the full image array in terms of basis-region images $\mathbf{b}^{^{\prime}}_{lk}$ as

\begin{equation*} \mathbf{b}_k = \sum_{l \in \Psi_k} \mathcal{R}^{^{\prime} \top}_l \mathbf{b}^{^{\prime}}_{lk},\end{equation*}

where $\mathcal{R}^{^{\prime}}_l$ is a matrix of size $I_l \times I$ obtained from diagonal matrix $\mathcal{R}_l$ by removing rows containing all zeros in $\mathcal{R}_l$; and $\Psi_k$ a set of indices *l*’s of all the basis regions containing basis material *k*. The transpose of matrix $\mathcal{R}^{^{\prime}}_l$ transforms basis-region images $\mathbf{b}^{^{\prime}}_{lk}$ of size *I_l_* within basis region *l* into an image on the full image array of size *I*.

We use vector $\mathbf{f}_m (\mathbf{b})$ of size *I* to denote the VMI at energy bin *m*, which can be written as $\mathbf{f}_m(\mathbf{b}) = \sum_{k = 1}^K \mu_{k {m}} \mathbf{b}_k$ in terms of basis images **b**_*k*_ and is thus a function of basis image **b**. Using this relationship, along with equation (2), we can re-express the VMI at energy bin *m* as

\begin{equation*} \mathbf{f}^{^{\prime}}_{m} \left(\mathbf{b}^{^{\prime}}\right) = \sum_{l = 1}^L \sum_{k \in \Phi_{l}} \mu_{k {m}} \mathcal{R}^{^{\prime} \top}_l \mathbf{b}^{^{\prime}}_{lk},\end{equation*}

where vector $\mathbf{f}^{^{\prime}}_{m} (\mathbf{b}^{^{\prime}})$ of size *I* denotes the VMI as a function of basis-region image $\mathbf{b}^{^{\prime}}_{lk}$. As mentioned, using equation (2), one can readily show $\mathbf{f}^{^{\prime}}_m(\mathbf{b}^{^{\prime}}) = \mathbf{f}_{m} (\mathbf{b})$.

##### 2.1.1.3. Possible reduction of unknowns

For discussion convenience, we introduce congregated basis-region image $\mathbf{b}^{^{\prime}}$ by concatenating $\mathbf{b}^{^{\prime}}_{lk}$ in the order of *k* and *l* and refer to $\mathbf{b}^{^{\prime}}$ simply as the basis-region image. It can be observed that the total number of voxel values, i.e. unknowns, in basis-region image $\mathbf{b}^{^{\prime}}$ is $N_\textrm{b} = \sum_{l = 1}^L K_l I_l$, which generally is smaller than $K I$, the total number of voxel values, i.e. unknowns, in basis image **b**. As mentioned above, assuming in the work that each basis region contains $K_l\unicode{x2A7D} 2$ known types of basis materials, we have $N_\textrm{b} \unicode{x2A7D} 2 I $ that is significantly smaller than $K I$, the total number of voxel values, i.e. unknowns, in $K\unicode{x2A7E} 3$ basis images defined on the full image array. Therefore, the introduction of basis regions can effectively reduce the total number of unknowns involved in the multi (i.e. $K\unicode{x2A7E} 3$)-basis images.

#### 2.1.2. Data augmentation with VC constraint

It has been previously investigated and observed that, in the case of $K_l\unicode{x2A7D} 2$, the basis-region-based data model in equation (5) can be inverted for accurate reconstruction of basis-region image $\mathbf{b}^{^{\prime}}$ directly from DE data (Chen *et al* 2024). However, it is highly unlikely to invert directly equation (5) for numerically accurate reconstruction of $\mathbf{b}^{^{\prime}}$ only from conventional data without imposing additional, adequate constraints. Therefore, we apply the VC constraint (Goodsitt *et al* 1987) on the basis-region images within basis region $l_c\in \Psi_{_\textrm{VC}}$ containing $K_{l_c} = 2$ known types of basis materials as

\begin{equation*} \textrm{VC}_{l_c}\left(\mathbf{b}^{^{\prime}}\right) = \sum_{k \in\Phi_{l_c }} \mathbf{b}^{^{\prime}}_{l_c k}/\rho_{k} - \mathbf{1}_{I_{l_c}} = 0,\end{equation*}

where $\Psi_{_\textrm{VC}}$ denotes a set of indices ${l_c}$ of basis regions to which the VC constraint is applied and $L_c = \mathrm{card}(\Psi_{_\textrm{VC}}) \unicode{x2A7D} L$; *ρ*_*k*_ depicts the known density of basis material *k*; and vector $\mathbf{1}_{I_{l_c}}$ is of size $I_{l_c}$ with entries of value 1. We note that no VC constraint needs to be applied to a basis region containing only $K_l = 1$ known type of basis materials such as air or metal. The total $\sum_{l_c} I_{l_c}$ explicit constraints in equation (4) are exploited for augmenting conventional data for accurate and stable reconstruction of basis-region images.

#### 2.1.3. Basis-region-based data model for image reconstruction

In order to reconstruct from conventional data the basis-region images with a reduced number of unknowns, a data model is needed that relates conventional data to basis-region images. Letting $g^{^{\prime}}_{j}(\mathbf{b}^{^{\prime}})$ denote model data in terms of basis-region image $\mathbf{b}^{^{\prime}}$, and substituting equation (2) into equation (1), we obtain (Chen *et al* 2024) the basis-region-based data model with reduced unknowns as

\begin{equation*} \begin{aligned} g^{^{\prime}}_{j}\left(\mathbf{b}^{^{\prime}}\right) &amp; = - \ln \sum_{m = 1}^{M} N_{jm}\left(\mathbf{b}^{^{\prime}}\right), \end{aligned}\end{equation*}

where

\begin{equation*} \begin{aligned} N_{jm}\left(\mathbf{b}^{^{\prime}}\right) &amp; = q_{j{m}} \exp \left( - \mathbf{A}_{j}^\top \sum_{l = 1}^L \sum_{k \in \Phi_{l }} \mu_{km} \mathcal{R}^{^{\prime} \top}_{l} \mathbf{b}^{^{\prime}}_{lk} \right). \end{aligned}\end{equation*}

Note that summations $\sum_{k = 1}^K \sum_{l \in \Psi_k}$ and $\sum_{l = 1}^L \sum_{k \in \Phi_l}$ are equivalent, as two ways to traverse the basis-region images over index *l* or *k* first. Again, as mentioned, using equations (1) and (2), one can readily show $g^{^{\prime}}_{j}(\mathbf{b}^{^{\prime}}) = g_{j}(\mathbf{b})$.

For discussion convenience below, we introduce vector $\mathbf{g}^{^{\prime}}(\mathbf{b}^{^{\prime}})$ of size *J* with entries $g^{^{\prime}}_{j}(\mathbf{b}^{^{\prime}})$ and then re-express (Chen *et al* 2024) the ray-based data model in equation (5) in a matrix-vector form as

\begin{equation*} \begin{aligned} \mathbf{g}^{^{\prime}}\left(\mathbf{b}^{^{\prime}}\right) = \mathcal{H}\left(\bar{\mathbf{b}}^{^{\prime}}\right) \mathbf{b}^{^{\prime}} + \Delta\mathbf{g}\left(\mathbf{b}^{^{\prime}}, \bar{\mathbf{b}}^{^{\prime}}\right), \end{aligned}\end{equation*}

where $\mathcal{H}(\bar{\mathbf{b}}^{^{\prime}}) \mathbf{b}^{^{\prime}}$ is the linear term when $\mathbf{g}^{^{\prime}}(\mathbf{b}^{^{\prime}})$ is expanded into the Taylor series at point $\bar{\mathbf{b}}^{^{\prime}}$ selected; matrix $\mathcal{H}(\bar{\mathbf{b}}^{^{\prime}})$ of size $J\times N_\textrm{b}$ has elements

\begin{equation*} h_{jlki^{^{\prime}}}\left(\bar{\mathbf{b}}^{^{\prime}}\right) = \frac{\sum_{m = 1}^{M} \mu_{km} N_{jm}\left(\bar{\mathbf{b}}^{^{\prime}}\right)}{\sum_{m = 1}^{M} N_{jm}\left(\bar{\mathbf{b}}^{^{\prime}}\right)} \sum_{i = 1}^I a_{ji} r^{^{\prime}}_{lii^{^{\prime}}};\end{equation*}

$r^{^{\prime}}_{lii^{^{\prime}}}$ is the element at row *i* and column *i*′ of matrix $\mathcal{R}^{^{\prime} \top}_l$. Note that equation (7) can simply be re-ordered to yield the defining expression for $\Delta\mathbf{g}(\mathbf{b}^{^{\prime}}, \bar{\mathbf{b}}^{^{\prime}})$ as

\begin{equation*} \Delta\mathbf{g}\left(\mathbf{b}^{^{\prime}}, \bar{\mathbf{b}}^{^{\prime}}\right) = \mathbf{g}^{^{\prime}}\left(\mathbf{b}^{^{\prime}}\right)-\mathcal{H}\left(\bar{\mathbf{b}}^{^{\prime}}\right) \mathbf{b}^{^{\prime}},\end{equation*}

which includes the non-linear component of the data model in equation (5). Therefore, our task is to invert equation (7) (or, equivalently, equation (5)) for numerically accurate reconstruction of basis-region images $\mathbf{b}^{^{\prime}}$ from which basis images **b**_*k*_ can readily be obtained by using equation (2).

### 2.2. Optimization-based image reconstruction

With the basis-region images of reduced number of unknowns in section 2.1.1 and VC constraint for data augmentation in section 2.1.2, the algorithm development is in order for numerically inverting the basis-region-based data model in equation (7) (or, equivalently, equation (5)) to achieve accurate and stable reconstruction of basis-region images from conventional data collected with a single spectrum in standard CT.

#### 2.2.1. Optimization program

We formulate the reconstruction problem of basis-region image $\mathbf{b}^{^{\prime}}$ (i.e. the inverse problem of equation (7)) from conventional data as a constrained optimization program:

\begin{align*} &amp;\mathbf{b}^{^{\prime}}* = \mathop{\text{arg min}}\limits_{\mathbf{b}^{^{\prime}}} \frac{1}{2} || \left[\mathbf{g}^{\left[\mathcal{M}\right]}- \Delta\mathbf{g}\left(\mathbf{b}^{^{\prime}}, \bar{\mathbf{b}}^{^{\prime}}\right) \right] - \mathcal{H}\left(\bar{\mathbf{b}}^{^{\prime}}\right) \mathbf{b}^{^{\prime}} ||_2^2 \nonumber\\ &amp;\text{s.t.} || \left(| \nabla \mathbf{f}^{^{\prime}}_{m_1} \left(\mathbf{b}^{^{\prime}}\right) |\right) ||_1 \unicode{x2A7D} \gamma_{m_1}, \,\, || \left(| \nabla \mathbf{f}^{^{\prime}}_{m_2} \left(\mathbf{b}^{^{\prime}}\right) |\right) ||_1 \unicode{x2A7D} \gamma_{m_2}, \nonumber\\ &amp; \qquad \quad \textrm{VC}_{l_c}\left(\mathbf{b}^{^{\prime}}\right) = 0 \,\, \text{for } l_c = 1,{\ldots},L_c, \nonumber\\ &amp; \qquad \quad \,\,\mathbf{b}^{^{\prime}} \succeq \mathbf{0}, \end{align*}

where vector $\mathbf{g}^{[\mathcal{M}]}$ of size *J* denotes conventional data measured with a single spectrum in a standard CT scan; $|| (|\nabla \mathbf{f}^{^{\prime}}_{m_i} (\mathbf{b}^{^{\prime}}) |) ||_1 $ the total-variation (TV) of VMI $\mathbf{f}^{^{\prime}}_{m_i}$ at energy bin *m_i_* selected; *L_c_* the number of basis regions subject to the VC constraint; and $\bar{\mathbf{b}}^{^{\prime}}$ is an input independent of $\mathbf{b}^{^{\prime}}$.

#### 2.2.2. Development of the dNCPD algorithm

The optimization program of interest in equation (10) is non-convex as a result of using the non-linear data model (i.e. equation (7)) in the objective function (i.e. line 1 in equation (10)). Because it is unclear if an algorithm can be developed for mathematically exactly converging the non-convex optimization program in equation (10), we take the approach, as described below and in Appendix A, to developing an algorithm to empirically converge the non-convex optimization program in equation (10) for numerically accurate and stable reconstruction of basis-region images (Chen *et al* 2021, 2024).

##### 2.2.2.1. The PD algorithm and its pseudo-codes

It can be shown that the non-convexity of equation (10) is caused by term $\Delta\mathbf{g}(\mathbf{b}^{^{\prime}}, \bar{\mathbf{b}}^{^{\prime}})$ that depends upon $\mathbf{b}^{^{\prime}}$, which is to be reconstructed. We can convexify equation (10) to obtain a convex optimization program in equation (A.1) in Appendix A.1 by replacing $\Delta\mathbf{g}(\mathbf{b}^{^{\prime}}, \bar{\mathbf{b}}^{^{\prime}})$ in equation (10) simply with constant $\overline{\Delta \mathbf{g}}$ that is independent $\mathbf{b}^{^{\prime}}$. One can thus derive a primal-dual (PD) algorithm to mathematically precisely solve the convex optimization program in equation (A.1) with its pseudo-codes shown in algorithm A1. We note that while the solution of equation (A.1) differs clearly from that of equation (10), the PD algorithm is derived to serve as a base for the development of the dNCPD algorithm, as discussed below and in Appendix A.2.

**Table:** 

**Algorithm A1.** Pseudo-codes of the PD algorithm for solving equation (A.1).
1: INPUT: $\mathbf{g}^{[\mathcal{M}]}$, $\gamma_{m_1}$, $\gamma_{m_2}$, *ρ*, and any $\bar{\mathbf{b}}^{^{\prime}}$ and $\overline{\Delta \mathbf{g}}$ independent of $\mathbf{b}^{^{\prime}}$
2: $\nu \leftarrow \frac{|| \mathcal{H}(\bar{\mathbf{b}}^{^{\prime}}) ||_2 }{|| \mathcal{I} ||_2 }$, $\alpha_1 \leftarrow \frac{|| \mathcal{H}(\bar{\mathbf{b}}^{^{\prime}}) ||_2}{|| \nabla \mathcal{U}_{m_1} ||_2}$, $\alpha_2 \leftarrow \frac{|| \mathcal{H}(\bar{\mathbf{b}}^{^{\prime}}) ||_2}{|| \nabla \mathcal{U}_{m_2} ||_2}$, $\beta_{l_c} \leftarrow \frac{|| \mathcal{H}(\bar{\mathbf{b}}^{^{\prime}}) ||_2}{|| \mathcal{V}_{l_c} ||_2}$
3: $\boldsymbol{\sigma} = (\boldsymbol{\sigma}_u^\top, \boldsymbol{\sigma}_v^\top, \boldsymbol{\sigma}_w^\top, \boldsymbol{\sigma}_p^\top, \boldsymbol{\sigma}_{q_{l_c}}^\top)^\top \leftarrow \frac{\rho}{|\mathcal{K}(\bar{\mathbf{b}}^{^{\prime}})| \mathbf{1}}$, $\boldsymbol{\tau} \leftarrow \frac{1}{\rho |\mathcal{K}(\bar{\mathbf{b}}^{^{\prime}})^\top| \mathbf{1}}$
4: $\displaystyle \Sigma_u \leftarrow \mathrm{diag}(\boldsymbol{\sigma}_u)$, $\Sigma_v \leftarrow \mathrm{diag}(\boldsymbol{\sigma}_v)$, $\Sigma_w \leftarrow \mathrm{diag}(\boldsymbol{\sigma}_w)$, $\Sigma_p \leftarrow \mathrm{diag}(\boldsymbol{\sigma}_p)$, $\Sigma_{q_{l_c}} \leftarrow \mathrm{diag}(\boldsymbol{\sigma}_{q_{l_c}})$, $\mathcal{T} \leftarrow \mathrm{diag}(\boldsymbol{\tau})$
5: INITIALIZE $\mathbf{b}^{^{\prime} (0)}, \mathbf{u}^{(0)}, \mathbf{v}^{(0)}, \mathbf{w}^{(0)}, \mathbf{p}^{(0)}$, and $\mathbf{q}_{l_c}^{(0)}$ to zeros
6: *n* = 0, $\tilde{\mathbf{b}}^{^{\prime} (0)} \leftarrow \mathbf{b}^{^{\prime} (0)}$
7: **repeat**
8: $\displaystyle \mathbf{u}^{(n+1)} = \frac{\mathbf{u}^{(n)} - \Sigma_u [(\mathbf{g}^{[\mathcal{M}]} - \overline{\Delta \mathbf{g}} ) - \mathcal{H}(\bar{\mathbf{b}}^{^{\prime}}) \tilde{\mathbf{b}}^{^{\prime} (n)}]} {1+\Sigma_u} $
9: $\mathbf{v}^{^{\prime} (n)} = \mathbf{v}^{(n)} + \Sigma_v \alpha_1 \nabla \mathcal{U}_{m_1} \tilde{\mathbf{b}}^{^{\prime} (n)} $
10: $\mathbf{w}^{^{\prime} (n)} = \mathbf{w}^{(n)} + \Sigma_w \alpha_2 \nabla \mathcal{U}_{m_2} \tilde{\mathbf{b}}^{^{\prime} (n)} $
11: $\displaystyle \mathbf{v}^{(n+1)} = \mathbf{v}^{^{\prime} (n)} - \Sigma_v \frac{\mathbf{v}^{^{\prime} (n)}}{|\mathbf{v}^{^{\prime} (n)}|} \mathrm{POL_1B}_{\alpha_1\gamma_{m_1}}\left(\frac{|\mathbf{v}^{^{\prime} (n)}|}{\Sigma_v} \right) $
12: $\displaystyle \mathbf{w}^{(n+1)} = \mathbf{w}^{^{\prime} (n)} - \Sigma_w \frac{\mathbf{w}^{^{\prime} (n)}}{|\mathbf{w}^{^{\prime} (n)}|} \mathrm{POL_1B}_{\alpha_2\gamma_{m_2}}\left( \frac{|\mathbf{w}^{^{\prime} (n)}|}{\Sigma_w} \right) $
13: $\mathbf{p}^{(n+1)} = \mathrm{neg}(\mathbf{p}^{(n)} + \Sigma_p \nu \tilde{\mathbf{b}}^{^{\prime} (n)} ))$
14: **for** $l_c = 1, \cdots, L_c$ **do**
15: $\mathbf{q}_{l_c}^{(n+1)} = \mathbf{q}_{l_c}^{(n)} + \Sigma_{q_{l_c}} \beta_{{l_c}} (\mathcal{V}_{l_c} \tilde{\mathbf{b}}^{^{\prime} (n)} - \mathbf{1}_{l_c})$
16: **end for**
17: $ \mathbf{b}^{^{\prime} (n+1)} = \mathbf{b}^{^{\prime} (n)} - \mathcal{T} [ \mathcal{H}(\bar{\mathbf{b}}^{^{\prime}})^\top\mathbf{u}^{(n+1)} + \alpha_1\mathcal{U}_{m_1}^\top\nabla^\top \mathbf{v}^{(n+1)} + \alpha_2\mathcal{U}_{m_2}^\top\nabla^\top\mathbf{w}^{(n+1)} + \nu \mathbf{p}^{(n+1)} + \sum_{l_c = 1}^{L_c} \beta_{l_c} \mathcal{V}^\top_{l_c} \mathbf{q}^{(n+1)}_{l_c} ] $
18: $ \tilde{\mathbf{b}}^{^{\prime} (n+1)} = 2\mathbf{b}^{^{\prime} (n+1)} - \mathbf{b}^{^{\prime} (n)} $
19: **until** convergence conditions are satisfied.

##### 2.2.2.2. The dNCPD algorithm and its pseudo-codes

It can be observed that while the optimization programs in equations (10) and (A.1) are mathematically different from each other, they appear to have identical forms. This observation thus motivates the development of the dNCPD algorithm by basing upon the PD algorithm, as described below and in Appendix A.2, to empirically converge the non-convex optimization program in equation (A.1), thus inverting the data model in equation (5) (or, equivalently, equation (1)) and possibly achieving numerically accurate and stable reconstruction of basis-region images, and subsequently of basis images and VMIs, from conventional data collected with a single spectrum.

In algorithm A2, we display the pseudo-codes of the dNCPD algorithm obtained by making a few changes to algorithm A1 of the PD algorithm, as described in Appendix A.2. Two key changes are made to the latter for obtaining the former: (i) $\mathbf{b}^{^{\prime} (n)}$ and $\mathcal{H}(\mathbf{b}^{^{\prime} (n)})$ are used as the estimates of $\bar{\mathbf{b}}^{^{\prime}}$ and $\mathcal{H}(\bar{\mathbf{b}}^{^{\prime}})$ and (ii) $\Delta \mathbf{g} (\mathbf{b}^{^{\prime} (n+1)}, {\mathbf{b}^{^{\prime} (n)}})$, which is computed with $\mathbf{b}^{^{\prime} (n+1)}$ and $\mathbf{b}^{^{\prime} (n)}$ in equation (9), as the estimate of $\overline{\Delta \mathbf{g}}$ in the iterative procedure, where *n* denotes the iteration number. The word ‘dynamic’ in the title of the dNCPD algorithm refers to the fact that the evaluation of linear system matrix $\mathcal{H}(\mathbf{b}^{^{\prime} (n)})$ is dependent on iteration number *n*. Comparison of algorithms A1 and A2 reveals that the iterative procedures in both pseudo-codes of the PD and dNCPD algorithms are of virtually identical iterative procedures, thus suggesting that the dNCPD algorithm may be of an empirical convergence behavior similar to that of the PD algorithm which is well characterized.

**Table:** 

**Algorithm A2.** Pseudo-codes of the dNCPD algorithm for empirically solving equation (10).
1: INPUT: $\mathbf{g}^{[\mathcal{M}]}$, $\gamma_{m_1}$, $\gamma_{m_2}$, *ρ*
2: $\nu \leftarrow \frac{|| \mathcal{H}(\bar{\mathbf{b}}^{^{\prime}}) ||_2 }{|| \mathcal{I} ||_2 }$, $\alpha_1 \leftarrow \frac{|| \mathcal{H}(\bar{\mathbf{b}}^{^{\prime}}) ||_2}{|| \nabla \mathcal{U}_{m_1} ||_2}$, $\alpha_2 \leftarrow \frac{|| \mathcal{H}(\bar{\mathbf{b}}^{^{\prime}}) ||_2}{|| \nabla \mathcal{U}_{m_2} ||_2}$, $\beta_{l_c} \leftarrow \frac{|| \mathcal{H}(\bar{\mathbf{b}}^{^{\prime}}) ||_2}{|| \mathcal{V}_{l_c} ||_2}$ with $\bar{\mathbf{b}}^{^{\prime}} = \mathbf{0}$
3: $\boldsymbol{\sigma} = (\boldsymbol{\sigma}_u^\top, \boldsymbol{\sigma}_v^\top, \boldsymbol{\sigma}_w^\top, \boldsymbol{\sigma}_p^\top, \boldsymbol{\sigma}_{q_{l_c}}^\top)^\top \leftarrow \frac{\rho}{|\mathcal{K}(\bar{\mathbf{b}}^{^{\prime}})| \mathbf{1}}$, $\boldsymbol{\tau} \leftarrow \frac{1}{\rho |\mathcal{K}(\bar{\mathbf{b}}^{^{\prime}})^\top| \mathbf{1}}$ with $\bar{\mathbf{b}}^{^{\prime}} = \mathbf{0}$
4: $\displaystyle \Sigma_u \leftarrow \mathrm{diag}(\boldsymbol{\sigma}_u)$, $\Sigma_v \leftarrow \mathrm{diag}(\boldsymbol{\sigma}_v)$, $\Sigma_w \leftarrow \mathrm{diag}(\boldsymbol{\sigma}_w)$, $\Sigma_p \leftarrow \mathrm{diag}(\boldsymbol{\sigma}_p)$, $\Sigma_{q_{l_c}} \leftarrow \mathrm{diag}(\boldsymbol{\sigma}_{q_{l_c}})$, $\mathcal{T} \leftarrow \mathrm{diag}(\boldsymbol{\tau})$
5: INITIALIZE $\mathbf{b}^{^{\prime} (0)}$, $\mathbf{u}^{(0)}, \mathbf{v}^{(0)}, \mathbf{w}^{(0)}, \mathbf{p}^{(0)}$, $\mathbf{q}_{l_c}^{(0)}$, and $\Delta \mathbf{g}^{(0)}$ to zeros
6: *n* = 0, $\tilde{\mathbf{b}}^{^{\prime} (0)} \leftarrow \mathbf{b}^{^{\prime} (0)}$
7: **repeat**
8: $\displaystyle \mathbf{u}^{(n+1)} = \frac{\mathbf{u}^{(n)} - \Sigma_u [(\mathbf{g}^{[\mathcal{M}]} - \Delta \mathbf{g}^{(n)} ) - \mathcal{H}(\mathbf{b}^{^{\prime} (n)}) \tilde{\mathbf{b}}^{^{\prime} (n)}]} {1+\Sigma_u} $
9: $\mathbf{v}^{^{\prime} (n)} = \mathbf{v}^{(n)} + \Sigma_v \alpha_1 \nabla \mathcal{U}_{m_1} \tilde{\mathbf{b}}^{^{\prime} (n)} $
10: $\mathbf{w}^{^{\prime} (n)} = \mathbf{w}^{(n)} + \Sigma_w \alpha_2 \nabla \mathcal{U}_{m_2} \tilde{\mathbf{b}}^{^{\prime} (n)} $
11: $\displaystyle \mathbf{v}^{(n+1)} = \mathbf{v}^{^{\prime} (n)} - \Sigma_v \frac{\mathbf{v}^{^{\prime} (n)}}{|\mathbf{v}^{^{\prime} (n)}|} \mathrm{POL_1B}_{\alpha_1\gamma_{m_1}}\left( \frac{|\mathbf{v}^{^{\prime} (n)}|}{\Sigma_v} \right) $
12: $\displaystyle \mathbf{w}^{(n+1)} = \mathbf{w}^{^{\prime} (n)} - \Sigma_w \frac{\mathbf{w}^{^{\prime} (n)}}{|\mathbf{w}^{^{\prime} (n)}|} \mathrm{POL_1B}_{\alpha_2\gamma_{m_2}}\left( \frac{|\mathbf{w}^{^{\prime} (n)}|}{\Sigma_w} \right) $
13: $\mathbf{p}^{(n+1)} = \mathrm{neg}(\mathbf{p}^{(n)} + \Sigma_p \nu \tilde{\mathbf{b}}^{^{\prime} (n)} ))$
14: **for** $l_c = 1, \cdots, L_c$ **do**
15: $\mathbf{q}_{l_c}^{(n+1)} = \mathbf{q}_{l_c}^{(n)} + \Sigma_{q_{l_c}} \beta_{{l_c}} (\mathcal{V}_{l_c} \tilde{\mathbf{b}}^{^{\prime} (n)} - \mathbf{1}_{l_c})$
16: **end for**
17: $ \mathbf{b}^{^{\prime} (n+1)} = \mathbf{b}^{^{\prime} (n)} - \mathcal{T} [ \mathcal{H}(\mathbf{b}^{^{\prime} (n)})^\top\mathbf{u}^{(n+1)} + \alpha_1\mathcal{U}_{m_1}^\top\nabla^\top \mathbf{v}^{(n+1)} + \alpha_2\mathcal{U}_{m_2}^\top\nabla^\top\mathbf{w}^{(n+1)} + \nu \mathbf{p}^{(n+1)} + \sum_{l_c = 1}^{L_c} \beta_{l_c} \mathcal{V}^\top_{l_c} \mathbf{q}^{(n+1)}_{l_c} ] $
18: $ \tilde{\mathbf{b}}^{^{\prime} (n+1)} = 2\mathbf{b}^{^{\prime} (n+1)} - \mathbf{b}^{^{\prime} (n)} $
19: $\Delta \mathbf{g}^{(n+1)} = \Delta \mathbf{g} (\mathbf{b}^{^{\prime} (n+1)}, {\mathbf{b}^{^{\prime} (n)}})$
20: $\Sigma_u = \mathrm{diag}\left( \frac{\rho}{|\mathcal{H}(\mathbf{b}^{^{\prime} (n+1)})| \mathbf{1}} \right), \mathcal{T} = \mathrm{diag}\left(\frac{1}{\rho |\mathcal{K}(\mathbf{b}^{^{\prime} (n+1)})^\top| \mathbf{1}}\right)$
21: **until** convergence conditions are satisfied.

While it remains unclear if the dNCPD algorithm can mathematically precisely solve the non-convex optimization program in equation (10), results of our extensive numerical studies, including those presented in section 3, indicate that the dNCPD algorithm can numerically accurately converge to the solution of the non-convex optimization program in equation (10). In the numerical studies in section 3, reconstruction of basis-region image $\mathbf{b}^{^{\prime}}$ is obtained when the convergence conditions defined in equation (B.6) in Appendix B are satisfied numerically in terms of the computer single-precision floating-point error. Numerically converged solution $\mathbf{b}^{^{\prime} *}$ in the pseudo-codes of the dNCPD algorithm is used as basis-region image $\mathbf{b}^{^{\prime}}$ reconstructed from which the basis images and VMIs can then be obtained by use of equations (2) and (3).

## 3. Numerical studies and results

Numerical studies are carried out in this work that are composed of a verification study and an evaluation study on the numerical accuracy and stability of the dNCPD algorithm. In the verification study, simulated data are generated from digital phantoms by use of non-linear data model equation (5) and thus are consistent completely with the data model; and they are thus used for verifying if the dNCPD algorithm can numerically accurately invert the non-linear data model in equation (5) through empirically converging the non-linear optimization program in equation (10). Meanwhile, in the evaluation study, real data of physical phantoms, which are collected by use of clinical DECT scanners, are inconsistent with the non-linear data model in equation (5) as they contain noise, scatter, and other physical factors. Therefore, real data are thus used for evaluating the stability of dNCPD algorithm in inverting the non-linear data model in equation (5) through empirically converging the non-convex optimization program in equation (10).

### 3.1. Verification study of the dNCPD algorithm

We first carry out a verification study to demonstrate that the dNCPD algorithm can empirically converge the non-convex optimization program in equation (10) for inverting the non-linear data model in equation (5) to reach numerically accurate reconstruction of multi ($K\unicode{x2A7E} 2$)-basis images, only from conventional data of a digital chest phantom.

#### 3.1.1. Verification study materials

##### 3.1.1.1. Digital chest phantom

**Figure 2.**
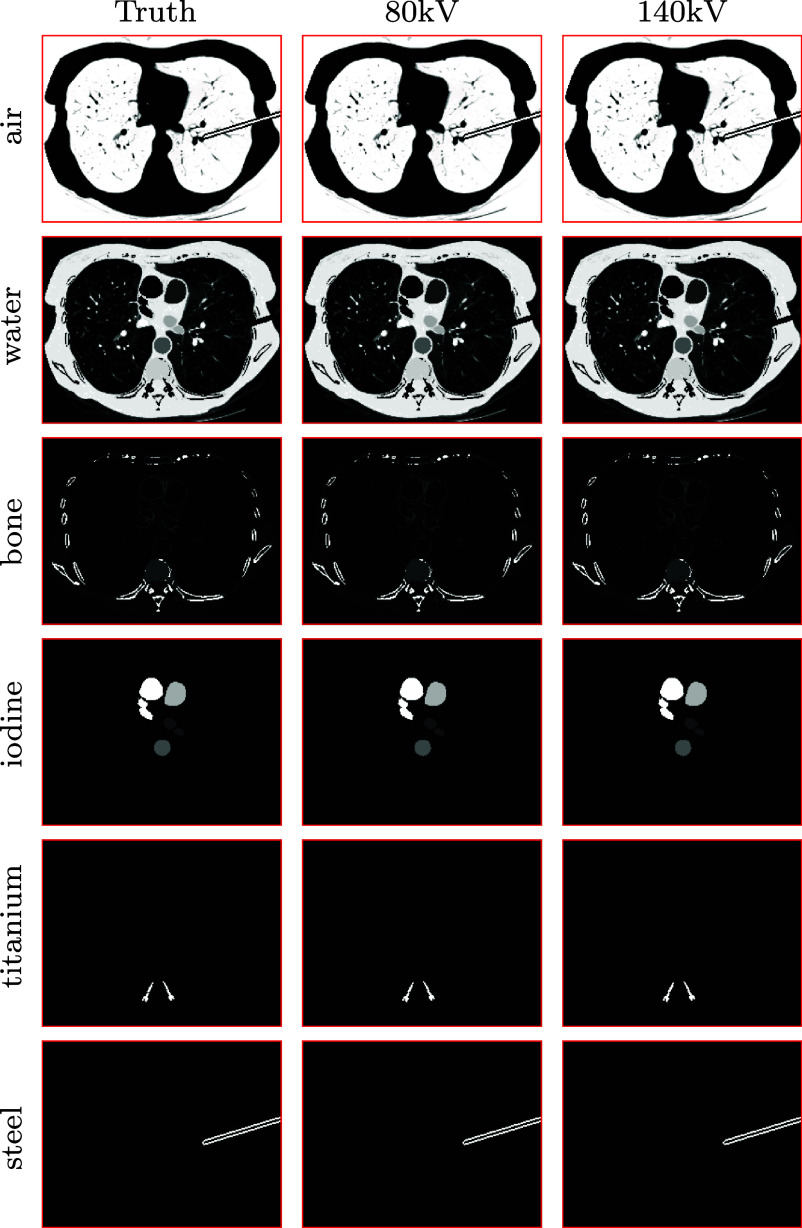
Truth basis images (column 1) of air (row 1), water (row 2), bone (row 3), 20 mg ml^−1^ iodine solution (row 4), titanium (row 5), and stainless steel (row 6) in the digital chest phantom; and basis images reconstructed by use of the dNCPD algorithm from simulated conventional data generated, respectively, using equation (5) with 80 kV (column 2) and 140 kV (column 3) spectra. Display windows: [0, 10^−3^] for basis image air and [0, 1] for other basis images.

The digital chest phantom contains a total of *K* = 6 basis materials, i.e. air, water, bone, 20 mg ml^−1^ iodine solution, titanium, and stainless steel; and a set of 5 spatially complementary basis regions, as depicted in figure 1, partitions the full image array of $200\times 256$ 0.14 cm square pixels. It is assumed that basis materials air, bone, 20 mg ml^−1^ iodine solution, titanium, and stainless steel are confined, respectively, within the 5 complementary basis regions shown in figure 1 from left to right, and basis material water distributes within the first three basis region, which is the full image array minus the regions containing titanium and air-steel. The titanium basis region (column 4) contains only the titanium basis material, while air is also allowed in the air-steel basis region (column 5), for the needle might contain air. Therefore, each basis region contains up to 2 basis materials. Furthermore, as the phantom includes *K* = 6 basis materials, it thus has *K* = 6 basis images on the full image array, referred to as the truth basis images, each of which contains only single basis material, as shown in column 1 of figure 2.

##### 3.1.1.2. Generation of simulated conventional data

Using the truth basis images in equation (5), we generate simulated, conventional data with each of two distinct spectra at 1440 views uniformly distributed over 2*π* with a circular fan-beam geometry that has a source-to-rotation (SOR) distance of 100 cm, a source-to-detector (SOD) distance of 150 cm, and a linear detector consisting of 896 detector bins of 0.08 cm bin size. Specifically, the two spectra of 80 kV and 140 kV are produced by use of the TASMIC package (Hernandez and Boone 2014), whereas the mass attenuation coefficients of the basis materials are either looked up from the NIST database (Hubbell and Seltzer 2004) or generated using the NIST XCOM tool (Berger *et al* 2010).

##### 3.1.1.3. Parameter selection

The dNCPD algorithm is used to reconstruct basis-region images from simulated conventional data of the digital chest phantom. In the reconstruction, geometrical and spectral information and truth basis regions used for the data generation are also used in the algorithm; whereas the TV-constraint parameters, $\gamma_{m_1}$ and $\gamma_{m_2}$ are computed from the truth VMIs at 50 and 100 keV, which can readily be obtained from the truth basis images in column 1 of figure 2.

#### 3.1.2. Verification study results

##### 3.1.2.1. Quantitative convergence results

**Figure 3.**
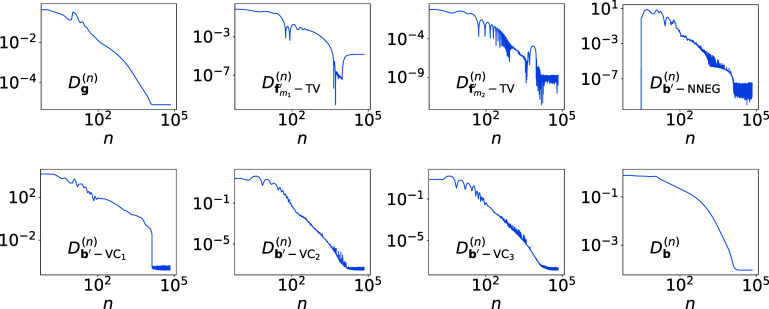
Convergence metrics of the dNCPD algorithm, defined by equations (B.1)–(B.4) (row 1, columns 1–4), equation (B.5) (row 2, columns 1–3), and equation (B.7) (row 2, column 4) in Appendix B, as functions of iteration *n*, from simulated 80 kV conventional data of the digital chest phantom in the verification study. (We note that equation (B.5) represents three metrics for the three basis regions considered in the study.) Similar convergence results, which are not shown here, are obtained also in the verification study using simulated 140 kV conventional data of the digital chest phantom.

In figure 3, we display the numerical convergence properties of the dNCPD algorithm in terms of convergence metrics, defined in Appendix B, as functions of iteration *n* from simulated conventional data generated with the 80 kV spectrum. It can be observed that the necessary convergence conditions in equation (B.6) are satisfied numerically in terms of the computer single-precision floating-point error, confirming that the dNCPD algorithm can empirically converge the non-convex optimization program in equation (10). Furthermore, as image-quality metric nRMSE, $D_{\textbf{b}}^{(n)}$, in the last panel in figure 3 shows, the condition in equation (B.8) on the reconstruction of basis images is satisfied numerically, verifying that for the conditions in the study, the dNCPD algorithm can numerically accurately invert the non-linear data model in equation (5) (or, equivalently, equation (1)). Similar convergence results are obtained for simulated conventional data generated with the 140 kV spectrum, thus they are not shown here.

##### 3.1.2.2. Basis images reconstructed

We also show in columns 2 and 3 of figure 2, basis images of air, water, bone, 20 mg ml^−1^ iodine solution, titanium, and stainless steel reconstructed, respectively, from simulated 80 and 140 kV conventional data. It can be observed that the basis images reconstructed from 80 and 140 kV data in columns 2 and 3 are visually identical to each other, and, more importantly, to the truth basis images in column 1 of figure 2, thus corroborating the convergence results in figure 3. The results of basis image reconstruction, along with the convergence evidence, verify that the dNCPD algorithm can numerically converge equation (10) for numerically accurate reconstruction of basis images, or, equivalently, numerically accurate inversion of the non-linear data model in equation (5) only from conventional data in standard CT.

In summary, in the verification study, metric nRMSE (i.e. the plot in panel (row 2 and column 4) of figure 3) and visual inspection (i.e. figure 2) are used to compare reconstructed images with their respective truth images, yielding the numerical evidence that the dNCPD algorithm can accurately invert the data model for achieving accurate image reconstruction. Other metrics, such as SSIM (Pambrun and Noumeir 2015), do not provide any more information than that by metric nRMSE on the convergence of the algorithm and the reconstruction accuracy of the images in the verification study, and hence are not included in the studies.

### 3.2. Study with real data

Once the dNCPD algorithm is verified for numerically accurate reconstruction of multi-basis images (or, equivalently, numerically accurate inversion of the non-linear data model in equation (5)) from simulated conventional data consistent with the data model, we then evaluate the stability of the dNCPD algorithm for reconstruction of multi-basis images from real conventional data, which contain necessarily physical factors, such as noise, scatter, and spectrum inaccuracy, that are not considered in, and thus inconsistent with, the non-linear data model in equation (5).

#### 3.2.1. Study materials of the physical DE phantom

##### 3.2.1.1. Physical DE phantom

In this real-data study, we use the widely-used, physical DE phantom that consists of a solid water disk with an approximate size of an average pelvis, 7 inserts of iodine contrast solutions at 2, 2.5, 5, 7.5, 10, 15, and 20 mg ml^−1^ concentrations, and 7 inserts of calcium solutions at 50, 100, 200, 300, 400, 500, and 600 mg ml^−1^ concentrations, as well as 8 air gap holes. The iodine and calcium inserts with various known concentration levels can be used for quantitative analysis including estimation of LAC and iodine concentration. The basis images and VMIs of the physical DE phantom are reconstructed on a full image array of $512 \times 512$ 0.08 cm square pixels.

##### 3.2.1.2. Real conventional data of the physical DE phantom

Real conventional data of the physical DE phantom were collected with 80 and 135 kV spectra by use of a clinical CT scanner (Chen *et al* 2024); and the scanner has multiple curved detector rows each of which consists of 896 equal-angular detector bins. The central curved detector row has SOR and SOD distances of 60 cm and 107 cm, thus forming a fan angle of 49^∘^. In the reconstruction studies below, we use real conventional data collected on the central curved detector row in axial mode at 1200 views evenly distributed over 2*π*. For the clinical scanner, we estimate the 80 and 135 kV spectra, respectively, for use in the image reconstruction (Chen *et al* 2024).

##### 3.2.1.3. Parameter selection

As discussed, the physical DE phantom consists of four (*K* = 4) types of basis materials, including air, water, 600 mg ml^−1^ calcium solution, and 20 mg ml^−1^ iodine solution; and we partition the full image array into three basis regions each of which contains two types of basis materials. It can be observed that the optimization program in equation (10) is specified by several parameters, including TV-constraint parameters $\gamma_{m_1}$ and $\gamma_{m_2}$ and also the selection of basis regions.

While knowledge of these parameters is known in the verification study in section 3.1 because the truth digital chest phantom is known, in a real-data study, the parameters need to be estimated generally empirically because their respective ‘truths’ are generally unknown or even undefined precisely. Instead, given a set of real conventional data, we perform multiple reconstructions with different sets of $\gamma_{m_1}$ and $\gamma_{m_2}$ and basis regions selected and then choose the set that yields the basis images of visually minimal artifacts for yielding the ‘optimal’ image reconstruction from real conventional data.

**Figure 4.**
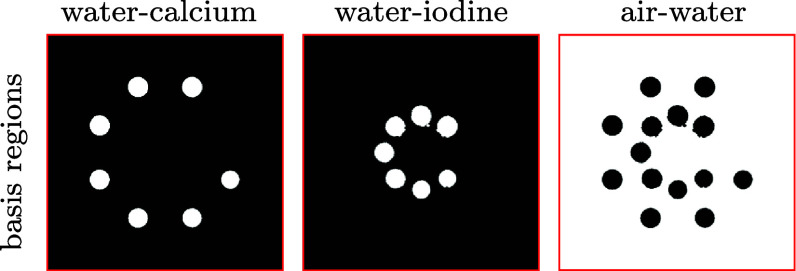
Basis regions selected for use in reconstruction of multi-basis images in the studies of the physical DE phantom. Each of the basis regions, highlighted in white, is displayed on the full image array and has a number of active pixels smaller than the number of pixels within the full image array. The basis regions contain water and 600 mg ml^−1^ calcium solution (column 1), water and 20 mg ml^−1^ iodine solution (column 2), and air and water (column 3).

**Figure 5.**
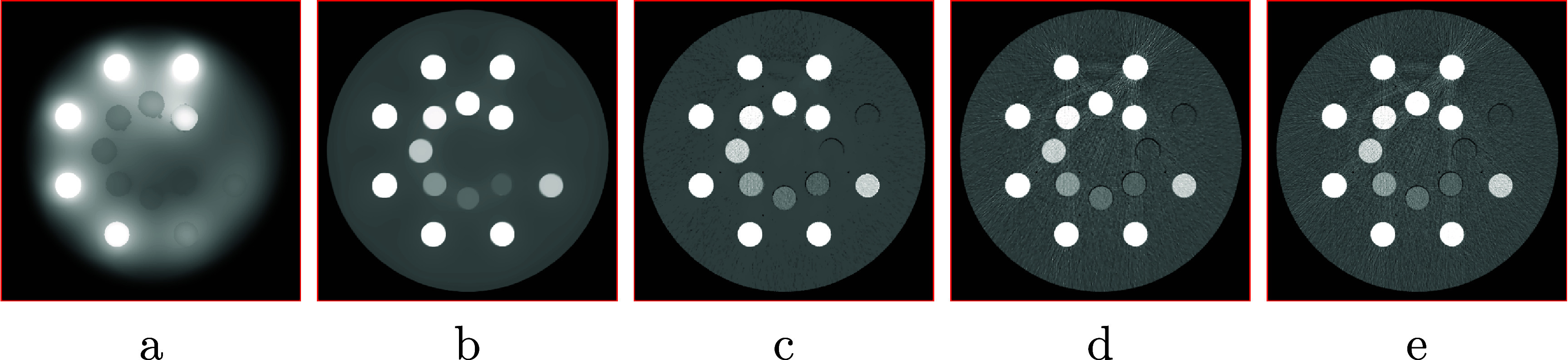
VMIs at 80 keV reconstructed from real, conventional data of the physical DE phantom collected with 80 kV spectra by use of the proposed dNCPD algorithm with TV-constraint parameter values $(\gamma_{m 1}, \gamma_{m 2}) = (247.0, 217.0)$ (a), (494.0, 434.0) (b), (741.0, 651.0) (c), (1111.5, 976.5) (d), and (1482.0, 1302.0) (e). Parameter values $(\gamma_{m 1}, \gamma_{m 2}) = (741.0, 651.0)$ in (c) are chosen as the ‘optimal’ selection, since the corresponding VMI is with the visually minimal artifacts. Display window: [−200, 200] HU.

In the physical DE-phantom study, the basis regions so selected are shown in figure 4. An example of selecting the TV-constraint parameters is shown in figure 5, where the VMIs at 80 keV are reconstructed from the real data of the physical DE phantom collected with the 80 kV spectra by the dNCPD algorithm with five sets of TV-constraint parameter values $\gamma_{m1}$ and $\gamma_{m2}$. It can be observed that the VMI in figure 5(c) (column 3) is with visually minimal artifacts, and thus its corresponding parameter values $(\gamma_{m 1}, \gamma_{m 2}) = (741.0, 651.0)$ are chosen as the optimal selection. The selection process of the TV-constraint parameters for the real data collected with the 135 kV spectra is exactly the same as described above and as illustrated for the one with the 80 kV spectra.

#### 3.2.2. Study results of the physical DE phantom

##### 3.2.2.1. Reconstruction of basis images and VMIs from conventional data

**Figure 6.**
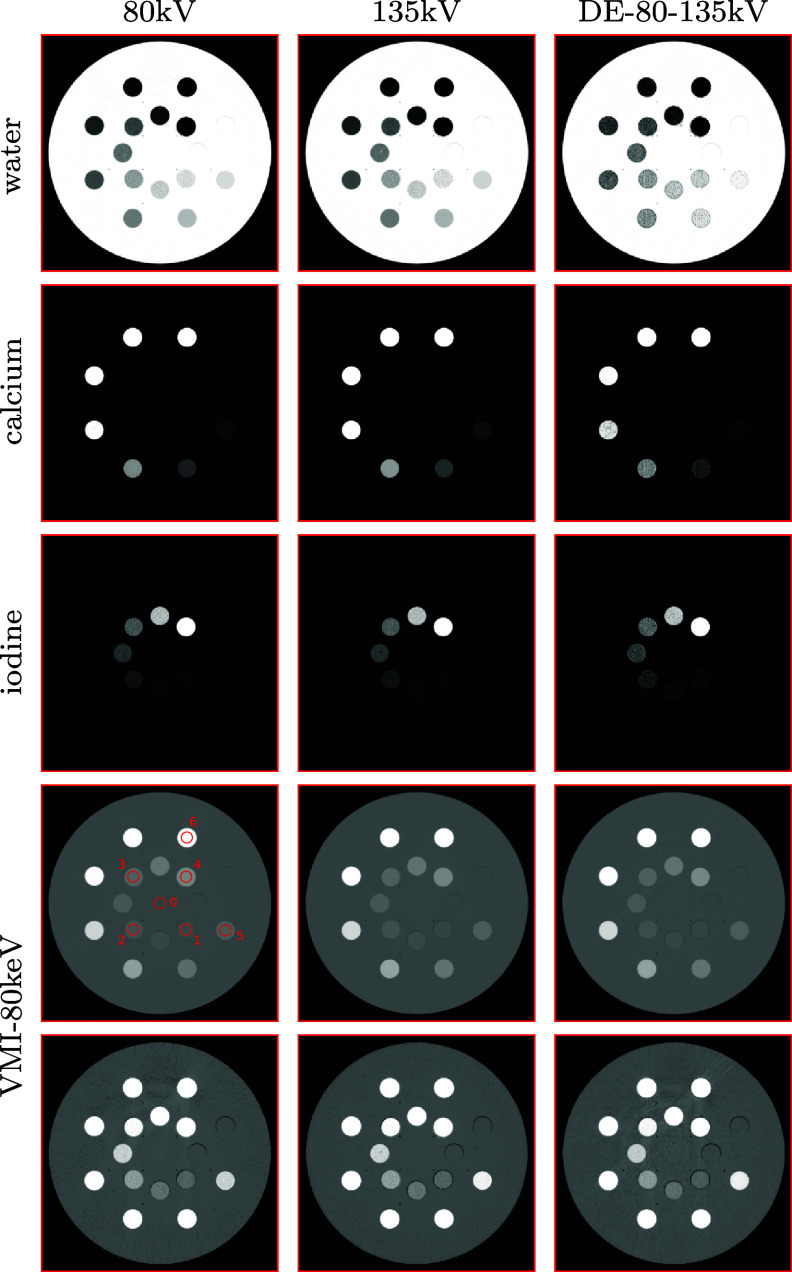
Basis images of water (row 1), 600 mg ml^−1^ calcium solution (row 2), and 20 mg ml^−1^ iodine solution (row 3), and VMIs at 80 keV (rows 4 and 5) reconstructed from real, conventional data of the physical DE phantom collected with 80 kV (column 1) and 135 kV (column 2) spectra by use of the dNCPD algorithm and from real DE-80-135kV data (column 3) by use of the existing algorithm (Chen *et al* 2024). The basis images of air reconstructed is not shown because it is numerically close to zero, containing only air gaps in the phantom. Display windows: [0, 1] for the basis images in rows 1–3, [−1000, 1000] HU for VMIs in row 4, and [−200, 200] HU for VMIs in row 5. The red circles in the panel, specified by row 4 and column 1, highlight the regions of interest (ROIs) selected for quantitative stability analysis of the dNCPD algorithm.

As described above, given each of conventional data sets collected with either 80 kV or 135 kV spectrum, we select the constraint parameters and the basis regions shown in figure 4, that are needed in the dNCPD algorithm for reconstruction of basis-region images. Using the basis-region images reconstructed in equations (2) and (3), we can readily obtain the basis images and then VMI. In figure 6, we display the basis images and VMI at 80 keV reconstructed, respectively, from 80 kV (column 1) and 135 kV (column 2) conventional data sets. It can be observed that the dNCPD algorithm can stably reconstruct multi-basis images and also VMIs directly from real conventional data collected with either 80 kV or 135 kV spectrum.

Conversely, the two sets of data collected with 80 and 135 kV spectra can also be used to form DE data, referred to as DE-80-135kV data, similar to that collected in DECT. Therefore, for comparison, we also apply an existing algorithm (Chen *et al* 2024) to reconstructing basis images and VMIs from DE-80-135kV data formed. In column 3 of figure 6, we display the basis images and VMI at 80 keV reconstructed from DE-80-135kV data. It can be observed in figure 6 that basis images and VMIs reconstructed from conventional data collected with either 80 kV or 135 kV spectrum appear to be visually comparable to the corresponding basis images and VMIs reconstructed from DE-80-135kV data.

##### 3.2.2.2. Quantitative analysis of the images reconstructed

In addition to visual inspection, we also conduct quantitative analysis of the basis images and VMIs reconstructed. We first quantitatively analyze estimates of iodine concentrations (ICs) within 6 regions-of-interest (ROIs) selected in figure 6, which center around solid water, iodine solutions of 2, 5, 10, and 20 mg ml^−1^, and calcium solution of 50 mg ml^−1^, respectively. Considering an affine relationship between the concentration level and the pixel values in the iodine basis image (Chen *et al* 2021), we can obtain IC values at the pixels in the iodine-basis image within the ROIs indicated in figure 6 and then average the IC values over each of the respective ROIs. Using the average ICs obtained, we then compute their biases and standard deviations (SDs) relative to the respective reference ICs provided by the manufacturer of the physical DE phantom, which are summarized in table 1. It can be observed that the biases and SDs of the IC estimates within the iodine basis images reconstructed from either 80 kV or 135 kV conventional data are comparable and that they are also comparable to those obtained from DE-80-135kV data. The results thus demonstrate quantitatively accuracy and stability of the dNCPD algorithm in reconstruction of the basis images from conventional data collected with a single spectrum in standard CT.

**Table 1.** Biases and SDs of iodine concentrations (ICs) estimated, relative to their respective reference ICs, within ROIs, labeled 0–5 in figure 6, containing water, iodine solutions of 2, 5, 10, and 20 mg ml^−1^, and calcium solution of 50 mg ml^−1^ in the iodine basis image, reconstructed from 80 kV (row 2) and 135 kV (row 3) conventional data by use of the dNCPD algorithm and from DE-80-135kV data (row 4) by use of an existing algorithm, respectively. The reference ICs provided by the manufacturer are depicted in parentheses in row 1.

	IC	water	iodine	iodine	iodine	iodine	calcium
	(mg ml^−1^)	(0)	(2)	(5)	(10)	(20)	(0)
80 kV	bias	0.08	0.10	0.20	0.58	0.01	0.08
	STD	0.0	0.62	0.78	0.89	2.7 × 10^−7^	0.0

135 kV	bias	0.11	0.01	0.74	0.12	0.00	0.09
	STD	0.0	0.89	0.94	1.1	1.3 × 10^−7^	0.0

DE	bias	0.30	0.34	0.06	0.47	0.04	0.30
	STD	0.0	2.0	2.7	3.1	0.50	0.0

**Figure 7.**
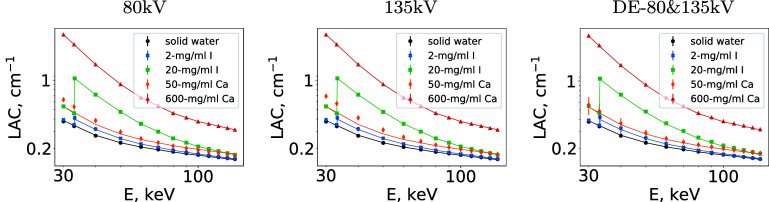
Averaged LACs over ROIs containing solid water (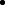), 2 mg ml^−1^ iodine (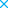), 20 mg ml^−1^ iodine (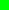), 50 mg ml^−1^ calcium (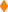), and 600 mg ml^−1^ calcium (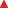) as functions of energy *E* within VMIs reconstructed from 80 kV conventional data (left), 135 kV conventional data (middle), and DE-80-135kV data (right). The corresponding SDs estimated are also shown as vertical bars on each markers, although some of the SDs might be too small to be visually discernible. The solid curves indicate reference LAC values computed by using the NIST XCOM package and the manufacturer’s spec sheet.

We also perform a quantitative analysis of the VMIs reconstructed over energy range $30\sim 140$ keV by computing average LACs, along with their respective SDs, over 5 ROIs, labeled 0, 1, 4, 5, and 6 in figure 6, containing solid water, iodine solutions of 2 and 20 mg ml^−1^, and calcium solutions of 50 and 600 mg ml^−1^. The LACs, along with their SDs, averaged over their respective ROIs, as displayed in figure 7, are estimated from VMIs reconstructed from 80 kV or 135 kV conventional data. It can be observed that the estimated LACs agree generally well with the respective reference values (solid curves in figure 7) computed from the manufacturer’s spec sheet. The results reveal quantitative accuracy and stability of the dNCPD algorithm in VMI reconstruction across the energy range from conventional data collected with a single spectrum in standard CT. It can also be observed that the results in the left and middle panels of figure 7 obtained from the conventional data appear to be comparable to those in the right panel of figure 7 obtained from the DE-80-135kV data.

#### 3.2.3. Study materials of the clinical abdomen phantom

##### 3.2.3.1. The clinical abdomen phantom

We also carry out an additional real-data study using conventional data collected from a clinical abdomen phantom with 80 kV or 135 kV spectrum. The clinical abdomen phantom is chosen because it possesses realistic anatomic complexity similar to that of a human abdomen. Considering the anatomic composition of the clinical abdomen phantom, four (*K* = 4) basis materials, including air, adipose, bone, and 20 mg ml^−1^ iodine solution, are selected for reconstructing multi (*K* = 4)-basis images directly from 80 kV or 135 kV conventional data. This study is used for further investigating the stability of dNCPD algorithm with respect to different levels of anatomic complexity and clinical relevance. The basis images and VMIs of the phantom are reconstructed on a full image array of $432\times 656$ 0.08 cm square pixels.

##### 3.2.3.2. Real conventional data collected

Real conventional data of the clinical abdomen phantom are collected with 80 and 135 kV spectra at 1200 views evenly distributed over 2*π* by use of the same clinical CT scanner as that in the study with the physical DE phantom in section 3.2.1. The 80 and 135 kV spectra estimated in section 3.2.1 are used also in image reconstruction of the clinical abdomen phantom.

##### 3.2.3.3. Parameter selection

**Figure 8.**
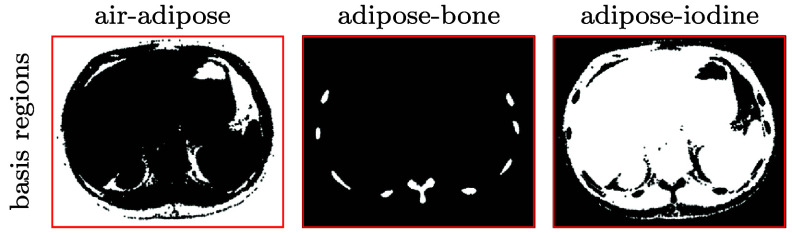
Basis regions selected for use in reconstruction of multi-basis images in the studies with real conventional data of the clinical abdomen phantom. Each of the basis regions, highlighted in white, is displayed on the full image array and has a number of active pixels smaller than the number of pixels within the full image array. The basis regions contain air and adipose (column 1), adipose and bone (column 2), and adipose and 20 mg ml^−1^ iodine solution (column 3).

Considering four (*K* = 4) types of basis materials, including air, adipose, bone, and 20 mg ml^−1^ iodine solution, we select basis regions that partition the full image array and assume two types of the basis materials within each of basis regions. We take the same approach as that described in section 3.2.1 to selecting TV-constraint parameters $\gamma_{m_1}$ and $\gamma_{m_2}$ and basis images. Namely, giving each set of real conventional data collected with 80 and 135 kV spectra, we perform multiple reconstructions with different sets of TV constraint parameters $\gamma_{m_1}$ and $\gamma_{m_2}$ and basis regions, and then choose the set that yields the basis images of visually minimal artifacts for use the final reconstruction of the basis images. In the study, the basis regions so chosen are shown in figure 8. The selection process of the TV-constraint parameters for the real data of the clinical abdomen phantom collected with the 80 and 135 kV spectra is exactly the same as that illustrated for the real data of the physical DE phantom with the 80 kV spectra in section 3.2.1 above.

#### 3.2.4. Study results of the clinical abdomen phantom

##### 3.2.4.1. Reconstruction of basis images and VMIs from conventional data

**Figure 9.**
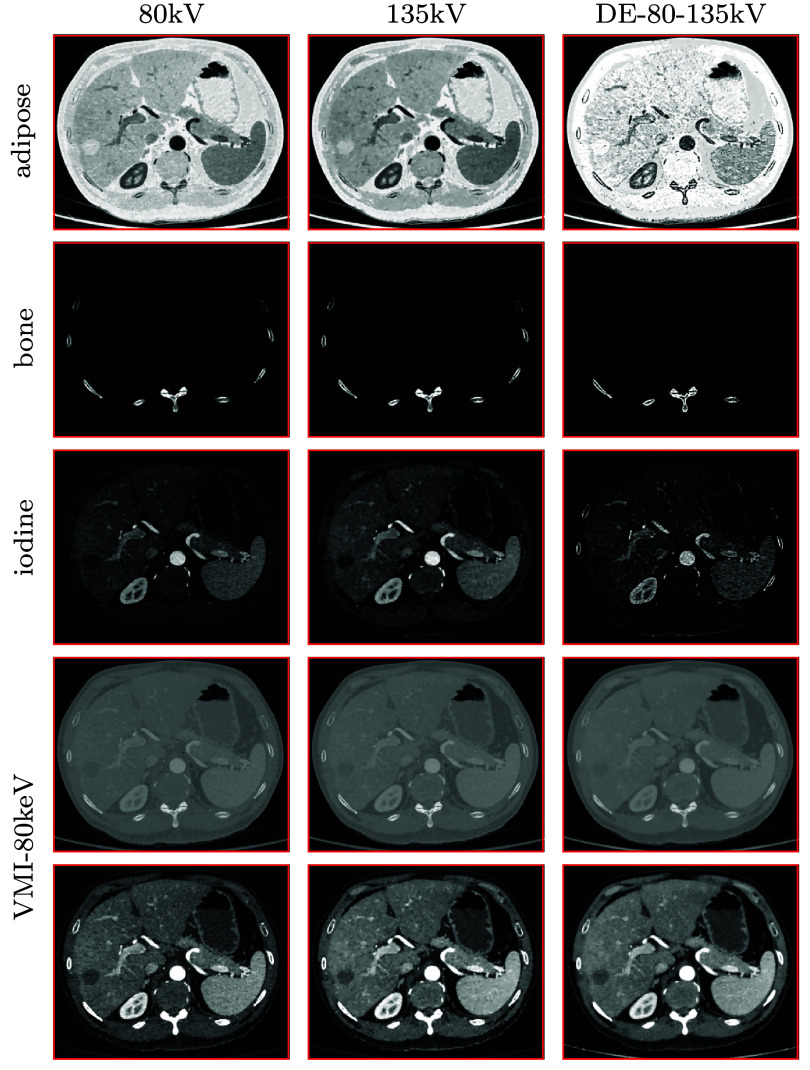
Basis images of adipose (row 1), bone (row 2), and 20 mg ml^−1^ iodine solution (row 3), and VMIs at 80 keV (rows 4 and 5) reconstructed from real 80 kV (column 1) and real 135 kV (column 2) conventional data of the clinical abdomen phantom by use of the dNCPD algorithm and from real DE-80-135kV data (column 3) by use of an existing algorithm (Chen *et al* 2024). The basis images of the air reconstructed is not shown because they are numerically close to zero, containing no structures. Display windows: [0, 1] for basis images in rows 1–3, [−1000, 1000] HU for VMIs in row 4, and [−160, 240] HU for VMIs in row 5.

As described above, for each set of 80 and 135 kV conventional data, we select the constraint parameters, including the basis regions shown in figure 8, for reconstruction of basis-region images by use of the dNCPD algorithm. Using the basis-region images reconstructed in equations (2) and (3), we can readily obtain the basis images and then VMI at 80 keV, which are displayed in figure 9. It can be observed that the dNCPD algorithm can stably reconstruct multi-basis images and also VMIs directly from real conventional data collected with either 80 kV or 135 kV spectrum. Because no knowledge of the ground truth of the clinical abdomen phantom is available, no quantitative analysis of the reconstruction in terms of IC and LAC estimations, similar to that in section 3.2.1, is performed.

Again, the two sets of data of the clinical abdomen phantom collected with 80 and 135 kV spectra can be used to form DE data, also referred to as DE-80-135kV data, similar to that collected in DECT. Therefore, for comparison, we also apply the same existing algorithm (Chen *et al* 2024) to reconstructing basis images and VMIs from DE-80-135kV data formed. In column 3 of figure 9, we display the basis images and VMI at 80 keV reconstructed from DE-80-135kV data. It can be observed in figure 9 that basis images and VMIs reconstructed from conventional data collected with either 80 kV or 135 kV spectrum appear to be visually comparable to the corresponding basis images and VMIs reconstructed from DE-80-135kV data.

In summary, in the real-data study, we conduct three types of comparison: (i) multiple images reconstructed with multiple sets of parameter values are visually compared (e.g. figure 5) for empirical selection of optimal parameter values; (ii) the images reconstructed from conventional single kV data are compared with the images reconstructed from the corresponding DE data (e.g. figures 6 and 9,) revealing that the images obtained from conventional, real data with the dNCPD algorithm are visually comparable to the corresponding standard DE images; and (iii) the IC and LAC estimates (e.g. table 1 and figure 7) of the physical DE phantom are compared with the respective reference values obtained using the NIST XCOM package and the manufacturer’s spec sheet.

## 4. Discussion

In the work, we develop the dNCPD algorithm to invert the non-linear data model in standard CT for numerically accurate and stable reconstruction of multi ($\unicode{x2A7E} \!\!2$)-basis images and VMIs directly from conventional data collected with a single spectrum in standard CT. The development of the dNCPD algorithm is enabled by the exploitation of the basis-region technique for reducing the number of voxels in basis images to be reconstructed and the VC constraint for effectively augmenting conventional data.

We conduct numerical studies on the performance of the dNCPD algorithm by using a qualitative metric of visual inspection and then quantitative metrics of (spatial-averaging) bias and variance of estimation of LACs and IC concentrations. Specifically, the accuracy of the dNCPD algorithm and its computer implementation are verified numerically first in a quantitative study with simulated conventional data of digital phantoms. Following the verification study, we evaluate and demonstrate the stability of the dNCPD algorithm for numerically accurate reconstruction of the basis images from real conventional data collected with a low- or a high-kVp spectrum in standard CT.

The derivation of the dNCPD algorithm in the work is similar to that in our prior work (Chen *et al* 2024) for reconstruction of multiple basis images from DECT data, but the dNCPD algorithm here is developed for reconstruction of multiple basis images only from a single set of conventional data from a standard CT scanner. The problem of image reconstruction (or, equivalently, of the inversion of the non-linear data model) is formulated as an optimization program in equation (10), which is non-convex as a consequence of its inclusion of the non-linear data model in CT imaging. In an attempt to empirically converging the non-convex optimization program, we first convexify it to obtain a convexified optimization program so that a PD algorithm can be derived for accurately solving it; and we then tailor the PD algorithm derived to obtain the dNCPD algorithm for empirically solving the non-convex optimization program.

As such, the pseudo-code structures of the dNCPD and PD algorithms are identical, except for the additional non-linear correction step involving $\Delta\mathbf{g}(\mathbf{b}^{^{\prime}}, \bar{\mathbf{b}}^{^{\prime}})$ in the former. This indeed also provides a convenient empirical check of the convergence of the dNCPD algorithm when monochromatic x-rays are considered. In this case, the non-linear data model turns into the standard linear data model, the non-convex optimization program degenerates into a convex optimization program, and the dNCPD algorithm thus becomes the PD algorithm. Furthermore, the convergence properties of the dNCPD algorithm, in terms of the convergence metrics shown in figure 3, are also similar to those of the PD algorithm under the same data conditions (Chen *et al* 2021).

The dNCPD algorithm developed can be applied to reconstructing numerically accurately and stably multi($\unicode{x2A7E} \!\!2$)-basis images only from conventional data assuming that each of the basis regions contains $K_l \unicode{x2A7D} 2$ types of basis materials. This assumption is generally reasonable because photoelectric effect and Compton scattering are the two dominant interaction mechanisms in the x-ray-energy range of standard diagnostic CT, indicating two degrees of freedom in the decomposition that thus allow for up to two types of basis materials in the basis region. Meanwhile, in the presence of K-edge materials, a representative basis material is included, such as 20 mg ml^−1^ iodine solution in the work, and basis regions are selected such that the iodine-solution basis material is contained with a low-attenuating basis material, such as water or adipose, to increase the expansion space afforded by the decomposition model in that basis region. On the other hand, one possible limitation of the proposed method could come from the $K_l \unicode{x2A7D} 2$ assumption, which means that there could be at most 2 basis materials in a basis region. For certain DECT imaging applications where an image voxel may need to be decomposed into 3 basis materials, such as fat, liver tissue, and blood for liver fat quantification (Hur *et al* 2014), the decomposition problem for those image voxels becomes essentially underdetermined with 3 unknowns and 2 data points (Tang and Ren 2021). As such, the proposed method may not yield stable reconstruction results with standard scans only using a single spectrum. Another possible limitation could be the reliance on the prior reconstructed image from the conventional data as the initialization of basis regions. Under conditions of incomplete data, such as sparse view or limited-angular range, reconstructed images from conventional data could suffer image artifacts, which could result in initialized basis regions far away from desired ones. As such, the parameter selection for basis regions might involve optimization-based reconstruction with adapted constraints and take longer iterations.

Parameter selection is critical to the performance of any reconstruction algorithms. In the work, the basis regions and TV-constraint parameters are selected as their respective truth in the simulated-data study and by surveying the parameter space based on the empirical metric of visualization in the real-data study. Some guidance in the parameter space search can be provided in order to reduce the search domain. For example, TV values can be computed from FBP-reconstructed images from either low-kV or high-kV conventional data and then used as reference values for searching the TV-constraint parameters. The FBP-reconstructed images can also be segmented using simple hard-thresholding to provide a reference for selection of the basis-region partition. However, unlike the case in DECT with the VC constraint (Chen *et al* 2024), some prior knowledge of the distribution of K-edge materials needs to be incorporated, as it might be challenging to separate iodine and bone based only upon pixel values.

Future investigation would include studies on the impact of other sources of inconsistencies, such as spectrum inaccuracy and scatter, on the performance of the dNCPD algorithm and studies involving patient data with specific clinically-related tasks, such as diagnosing bone edema (Schwaiger *et al* 2018) and iodine contrast quantification in breast cancer staging (Volterrani *et al* 2020). The dNCPD algorithm can readily be generalized to reconstruct basis images and VMIs from conventional data collected with standard cone-beam CT (Siewerdsen *et al* 2005) and be applied directly to conventional data collected at sparse views in fan- and cone-beam CT (Bian *et al* 2010) as the non-convex optimization program and the dNCPD algorithm are formulated in terms of the geometry of individual x-rays in fan- and cone-beam CT. It would also be interesting to tailor the dNCPD algorithm by including constraints on, e.g. image directional TVs, to address the reconstruction problem of basis images and VMIs from conventional data collected only over a limited-angular range (Zhang *et al* 2021). Lastly, the stability of the proposed method can be further investigated by applying it to data collected with known 3 basis materials, as mentioned above, where the assumption of at most 2 basis materials in a basis region is not valid. Such investigations can generate insights into the depth and width of the potential practical implications of the proposed method.

## 5. Conclusions

We develop, verify, and evaluate the dNCPD algorithm that numerically accurately and stably reconstructs $K\unicode{x2A7E} 2$ basis images and VMIs directly from conventional data collected only with a single spectrum in standard CT. The work is of potential clinical relevance as it reveals that, using conventional data, the dNCPD algorithm yields images of quality comparable to that of images reconstructed from data in DECT that has been adopted for clinical and other applications. The work is also of technical significance and novelty as it yields, through integrating the VC constraint and the basis-region technique, for the first time an algorithm, i.e. the dNCPD algorithm, for numerically accurately inverting the non-linear data model to achieve multi-basis image reconstruction only from conventional data in standard CT.

The algorithm can potentially be exploited for reducing imaging time and dose, and/or system cost in DECT, as the work reveals the possibility of obtaining multi-basis images and VMIs only from conventional data in standard CT, instead of data collected in DE, multi-spectra, or photon-counting CT, which generally involve either additional scanning effort and/or additional, unique hardware components/systems. Furthermore, because the ray-based non-linear data model in equation (1) can readily accommodate x-rays in any projection geometries, including cone-beam and fan-beam geometries, the dNCPD algorithm can readily be extended to reconstruction of multi-basis images and VMIs from conventional data collected in fan- and cone-beam CT at sparse views or over a limited-angular range.

## Data Availability

The data cannot be made publicly available upon publication because the cost of preparing, depositing and hosting the data would be prohibitive within the terms of this research project. The data that support the findings of this study are available upon reasonable request from the authors.

## Appendix A The dNCPD algorithm

### A.1. The PD algorithm and its pseudo-codes

As illustrated in sections 2.1.3 and 2.2.2, using constant term $\overline{\Delta \mathbf{g}}$ to replace $\Delta\mathbf{g}({\mathbf{b}}^{^{\prime}}, \bar{\mathbf{b}}^{^{\prime}})$ in equation (10), we then obtain

\begin{equation*} \begin{aligned} \mathbf{b}^{^{\prime} *} &amp; = \mathop{\text{arg min}}\limits_{\mathbf{b}^{^{\prime}}} \frac{1}{2} || \left[\mathbf{g}^{\left[\mathcal{M}\right]}- \overline{\Delta\mathbf{g}}\right] - \mathcal{H}\left(\bar{\mathbf{b}}^{^{\prime}}\right) \mathbf{b}^{^{\prime}} ||_2^2 \\ \text{s.t.} \,\,&amp; || \left(| \nabla \mathbf{f}^{^{\prime}}_{m_1} \left(\mathbf{b}^{^{\prime}}\right) |\right) ||_1 \unicode{x2A7D} \gamma_{m_1}, \,\, || \left(| \nabla \mathbf{f}^{^{\prime}}_{m_2} \left(\mathbf{b}^{^{\prime}}\right) |\right) ||_1 \unicode{x2A7D} \gamma_{m_2},\\ &amp; \textrm{VC}_{l_c}\left(\mathbf{b}^{^{\prime}}\right) = 0 \,\, \text{for } l_c = 1,{\ldots},L_c, \\ &amp; \mathbf{b}^{^{\prime}} \succeq \mathbf{0}. \end{aligned}\end{equation*}

Because $\overline{\Delta \mathbf{g}}$ and $\bar{\mathbf{b}}^{^{\prime}}$ (thus $\mathcal{H}(\bar{\mathbf{b}}^{^{\prime}})$) are selected to be independent of $\mathbf{b}^{^{\prime}}$, the optimization program in equation (A.1) is thus convex (with respect to ${\mathbf{b}}^{^{\prime}}$). As the general PD algorithm has been shown to converge any convex optimization (Rockafellar 1997, Chambolle and Pock 2010), we can then develop a PD algorithm (Sidky *et al* 2012), as an instance of the general PD algorithm, to converge the specific convex optimization program in equation (A.1), with its pseudo-codes displayed in algorithm A1. We note that the optimization program in equation (A.1) is convex with respect to $\mathbf{b}^{^{\prime}}$, as long as $\bar{\mathbf{b}}^{^{\prime}}$ and $\overline{\Delta \mathbf{g}}$ selected are independent of $\mathbf{b}^{^{\prime}}$, including a selection of $\bar{\mathbf{b}}^{^{\prime}} = \mathbf{0}$ and $\overline{\Delta \mathbf{g}} = \mathbf{0}$. It is worthy also to point out that $\bar{\mathbf{b}}^{^{\prime}}$ and $\overline{\Delta \mathbf{g}}$ selected remain unchanged through out the iterative procedure in algorithm A1.

Furthermore, in the pseudo-codes of algorithm A1, initial estimate $\mathbf{b}^{^{\prime} (0)} = \mathbf{0}$; $\mathbf{u}, \mathbf{v}, \mathbf{w}, \mathbf{p}$, and $\mathbf{q}_{l_c}$ are dual variables corresponding to the linear transforms in the primal problem for the objective function, TV constraints at *m*_1_ and *m*_2_, non-negativity constraint, and VC constraint for basis region *l_c_*, respectively; and vertically stacked matrix $\mathcal{K}(\bar{\mathbf{b}}^{^{\prime}})$ is given by

\begin{equation*} \begin{aligned} \mathcal{K}(\bar{\mathbf{b}}^{^{\prime}}) &amp;= \left(\mathcal{H}(\bar{\mathbf{b}}^{^{\prime}})^\top, \, \alpha_1 \left(\nabla \mathcal{U}_{m_1}\right)^\top, \alpha_2 \left(\nabla \mathcal{U}_{m_2}\right)^\top, \right.\\ &amp;\left. \nu \mathcal{I}, \beta_{1} \mathcal{V}_1^\top, {\ldots}, \beta_{L_c} \mathcal{V}_{L_c}^\top \right)^\top, \end{aligned}\end{equation*}

where linear transform matrix $\mathcal{H}(\bar{\mathbf{b}}^{^{\prime}})$ has elements $h_{jlki^{^{\prime}}}(\bar{\mathbf{b}}^{^{\prime}})$ defined in equation (8); matrix $\mathcal{U}_{m_i}$ of size $I \times N_\textrm{b}$ is given by

\begin{equation*} \begin{aligned} \mathcal{U}_{m_i} &amp; = \left(\mathcal{U}^{^{\prime}}_{1m_i},\mathcal{U}^{^{\prime}}_{2m_i},\cdots,\mathcal{U}^{^{\prime}}_{Lm_i}\right), \end{aligned}\end{equation*}

which transforms basis-region images to VMI at energy bin *m_i_* according to equation (3), where $\mathcal{U}^{^{\prime}}_{lm_i} = (\mathcal{R}^{^{\prime} \top}_l \mu_{k_1 m_i}, \mathcal{R}^{^{\prime} \top}_l \mu_{k_2 m_i}, \cdots, \mathcal{R}^{^{\prime} \top}_l \mu_{k_{K_l} m_i})$ and $m_i = m_1$ or *m*_2_; and lastly, each of a total of *L_c_* matrices $\mathcal{V}_{l_c}$ of size $I_{l_c} \times N_\textrm{b}$ is given by

\begin{equation*} \begin{aligned} \mathcal{V}_{l_c} &amp; = \left(\mathbf{0}, {\ldots}, \mathcal{I}_{l_c}/\rho_{k_1}, \mathcal{I}_{l_c}/\rho_{k_2}, {\ldots}, \mathcal{I}_{l_c}/\rho_{k_{K_{l_c}}}, {\ldots}, \mathbf{0}\right), \end{aligned}\end{equation*}

where $l_c\in \Psi_{_\textrm{VC}}$; $\mathcal{I}_{l_c}$ is an identity matrix of size $I_{l_c}$ and blocks corresponding to basis regions other than *l_c_* are all zeros **0** of size $I_{l_c} \times I_l K_l$ for $l \neq l_c$. Moreover, scaling factors *α*_1_, *α*_2_, *ν*, and $\beta_{l_c}$ are computed as the ratios of the largest singular values, as indicated by the $|| \cdot ||_2$ operator, of matrix $\mathcal{H}(\bar{\mathbf{b}}^{^{\prime}})$ and matrices $\mathcal{U}_{m_1}$, $\mathcal{U}_{m_2}$, $\mathcal{I}$, and $\mathcal{V}_{l_c}$, respectively (line 2 in algorithm A1); and $\Sigma_u, \Sigma_v, \Sigma_w, \Sigma_p, \Sigma_{q_{l_c}}$, and $\mathcal{T}$ are diagonal preconditioners for the PD algorithm, whose diagonal elements form vectors $\boldsymbol{\sigma}_u, \boldsymbol{\sigma}_v, \boldsymbol{\sigma}_w, \boldsymbol{\sigma}_p, \boldsymbol{\sigma}_{q_{l_c}}$, and ***τ***, which are computed (Chen *et al* 2024) by forwarding and back-projecting the absolute value of $\mathcal{K}(\bar{\mathbf{b}}^{^{\prime}})$ in equation (A.2) as

\begin{equation*} \begin{aligned} \Sigma_u &amp; = \mathrm{diag}\left(\boldsymbol{\sigma}_u\right) = \mathrm{diag}\left(\frac{\rho}{|\mathcal{H}\left(\bar{\mathbf{b}}^{^{\prime}}\right)| \mathbf{1}} \right), \\ \Sigma_v &amp; = \mathrm{diag}\left(\boldsymbol{\sigma}_v\right) = \mathrm{diag}\left(\frac{\rho}{|\alpha_1 \left(\nabla \mathcal{U}_{m_1}\right)| \mathbf{1}} \right), \\ \Sigma_w &amp; = \mathrm{diag}\left(\boldsymbol{\sigma}_w\right) = \mathrm{diag}\left(\frac{\rho}{|\alpha_2 \left(\nabla \mathcal{U}_{m_2}\right)| \mathbf{1}} \right), \\ \Sigma_p &amp; = \mathrm{diag}\left(\boldsymbol{\sigma}_p\right) = \mathrm{diag}\left(\frac{\rho}{|\nu \mathcal{I} | \mathbf{1}}\right), \\ \Sigma_{q_{l_c}} &amp; = \mathrm{diag}\left(\boldsymbol{\sigma}_{q_{l_c}}\right) = \mathrm{diag}\left(\frac{\rho}{|\beta_{l_c} \mathcal{V}_{l_c} | \mathbf{1}} \right), \\ \mathcal{T} &amp; = \mathrm{diag}\left(\boldsymbol{\tau}\right) = \mathrm{diag}\left(\frac{1}{\rho |\mathcal{K}\left(\bar{\mathbf{b}}^{^{\prime}}\right)^\top| \mathbf{1}}\right). \end{aligned}\end{equation*}

We note again that the PD algorithm, which converges the convex optimization problem in equation (A.1), is developed to serve as a base for the subsequent development of the dNCPD algorithm in Appendix A.2 for empirically converging the non-convex optimization program in equation (10) of our interest.

### A.2. The dNCPD algorithm and its pseudo-codes

As mentioned, in the pseudo-codes of the PD algorithm in algorithm A1, $\bar{\mathbf{b}}^{^{\prime}}$ and $\overline{\Delta \mathbf{g}}$, which are selected to be independent $\mathbf{b}^{^{\prime}}$, remain unchanged throughout the iterative procedure; and the PD algorithm thus cannot converge the non-convex program in equation (10). Instead, in an attempt to obtain the dNCPD algorithm for empirically converging equation (10), we tailor the PD algorithm, as described below, by replacing $\bar{\mathbf{b}}^{^{\prime}}$ and $\overline{\Delta \mathbf{g}}$ with their iteration-dependent estimates.

Specifically, the pseudo-codes of the dNCPD algorithm are obtained by making changes to the pseudo-codes of the PD algorithm, including: (i) in lines 2 and 3, using $\bar{\mathbf{b}}^{^{\prime}} = \mathbf{0}$ as the estimate of $\bar{\mathbf{b}}^{^{\prime}}$ in the computation of the parameters and diagonal preconditioners; (ii) in line 5, using $\Delta \mathbf{g}^{(0)} = \mathbf{0}$ as the initial estimate of $\overline{\Delta \mathbf{g}}$; (iii) in lines 8 and 17, using $\mathbf{b}^{^{\prime} (n)}$ and $\mathcal{H}(\mathbf{b}^{^{\prime} (n)})$ as the estimates of $\bar{\mathbf{b}}^{^{\prime}}$ and $\mathcal{H}(\bar{\mathbf{b}}^{^{\prime}})$; (iv) adding line 19 in algorithm A2 that uses $\Delta \mathbf{g}^{(n+1)} = \Delta \mathbf{g} (\mathbf{b}^{^{\prime} (n+1)}, {\mathbf{b}^{^{\prime} (n)}})$, which is computed with $\mathbf{b}^{^{\prime} (n+1)}$ and $\mathbf{b}^{^{\prime} (n)}$ in equation (9), as the estimate of $\overline{\Delta \mathbf{g}}$ in the iterative procedure; and (v) adding line 20 in algorithm A2 for dynamically updating the diagonal preconditioners (Chen *et al* 2021, 2024).

As all of the variables in algorithm A2 are identical to those defined in algorithm A1, it can be observed that the pseudo-codes of the PD and dNCPD algorithms are of virtually identical iterative procedures, thus suggesting that the dNCPD algorithm may be of an empirical convergence behavior similar to that of the PD algorithm which is well-characterized.

## Appendix B Convergence conditions

The necessary convergence conditions are designed to empirically verify that the dNCPD algorithm can numerically converge the non-convex optimization program in equation (10). As such, we first define the following dimensionless convergence metrics to track the evolution of the objective function and constraints in equation (10) as

\begin{align*} &amp; D_\mathbf{g}^{\left(n\right)} = || [ \mathbf{g}^{[\mathcal{M}]} - \mathbf{g}^{^{\prime}}\left(\mathbf{b}^{^{\prime} \left(n\right)}\right) ||_2 / || \mathbf{g}^{[\mathcal{M}]} ||_2,\end{align*}

\begin{align*} &amp; D_{\mathbf{f}^{^{\prime}}_{m_1}{\text{-}}\mathrm{TV}}^{\left(n\right)} = | \left(|| \nabla \mathcal{U}_{m_1} \mathbf{b}^{^{\prime} \left(n\right)} ||_1 - \gamma_{m_1} \right)| / \gamma_{m_1},\end{align*}

\begin{align*} &amp; D_{\mathbf{f}^{^{\prime}}_{m_2}{\text{-}}\mathrm{TV}}^{\left(n\right)} = | \left(|| \nabla \mathcal{U}_{m_2} \mathbf{b}^{^{\prime} \left(n\right)} ||_1 - \gamma_{m_2} \right)| / \gamma_{m_2},\end{align*}

\begin{align*} &amp; D_{\mathbf{b}^{^{\prime}}{\text{-}}\mathrm{NNEG}}^{\left(n\right)} = || \mathrm{neg}\left(\mathbf{b}^{^{\prime} \left(n\right)}\right) ||_2,\end{align*}

\begin{align*} &amp; D_{\mathbf{b}^{^{\prime}}\text{-}\mathrm{VC}_{l_c}}^{\left(n\right)} = || \mathrm{VC}\left(\mathbf{b}^{^{\prime} \left(n\right)}\right) ||_2, \,\, \text{for } l_c = 1,{\ldots},L_c.\end{align*}

The necessary convergence conditions are then defined as

\begin{equation*} \begin{aligned} &amp; D_\mathbf{g}^{\left(n\right)} \rightarrow \epsilon,\\ &amp; D_{\mathbf{f}^{^{\prime}}_{m_1}{\text{-}}\mathrm{TV}}^{\left(n\right)} \rightarrow 0 \,\,\, \text{and } \, D_{\mathbf{f}^{^{\prime}}_{m_2}{\text{-}}\mathrm{TV}}^{\left(n\right)} \rightarrow 0, \\ &amp; D_{\mathbf{b}^{^{\prime}}{\text{-}}\mathrm{NNEG}}^{\left(n\right)} \rightarrow 0, \\ &amp; D_{\mathbf{b}^{^{\prime}}{\text{-}}\mathrm{VC}_{l_c}}^{\left(n\right)} \rightarrow 0, \,\, \text{for } l_c = 1,{\ldots},L_c, \end{aligned}\end{equation*}

where $n\rightarrow \infty$; and constant *ε* = 0 in the consistent simulated-data study or *ε* > 0 in the inconsistent simulated- and real-data study.

We also introduce an image-quality metric that measures the difference between reconstructed basis image $\mathbf{b}^{(n)}$ and reference basis image $\mathbf{b}^{\mathrm{[ref]}}$, if available, as

\begin{align*} &amp; D_{\mathbf{b}}^{\left(n\right)} = || \mathbf{b}^{\left(n\right)} - \mathbf{b}^{\mathrm{[ref]}} ||_2 / || \mathbf{b}^{\mathrm{[ref]}} ||_2,\end{align*}

where basis image $\mathbf{b}^{(n)}$ on the full image array can be computed from the basis-region images using equation (2)

In the verification study (see, e.g. section 3.1) in which truth basis image $\mathbf{b}^{\mathrm{[truth]}}$ is known and simulated data are consistent with the data model in equation (5), we choose truth basis image $\mathbf{b}^{\mathrm{[truth]}}$ as reference $\mathbf{b}^{\mathrm{[ref]}}$ and thus use the image-quality metric to devise a sufficient convergence condition

\begin{equation*} D_{\textrm{b}}^{\left(n\right)} \rightarrow 0, \quad \textrm{as} \quad n\rightarrow \infty\end{equation*}

on the accuracy of reconstructing the basis images (or, equivalently, of inverting the non-linear data model in equation (5).)

We note that one may seek to evaluate the stability of the dNCPD algorithm in reconstruction of basis images by using real data (see, e.g. section 3.2) or simulated data containing physical factors inconsistent with the data model in equation (5). In this case, even if one can select $\mathbf{b}^\textrm{[ref]}$ to compute the metric in equation (B.7), condition equation (B.8) is generally not a sufficient and necessary condition on the numerically accurate inversion of data model equation (5) by use of the dNCPD algorithm; instead, it simply indicates that $\mathbf{b}^{(n)}$ approaches $\mathbf{b}^\textrm{[ref]}$.
